# Advertising of foods and beverages in social media aimed at children: high exposure and low control

**DOI:** 10.1186/s12889-022-14196-4

**Published:** 2022-09-22

**Authors:** Lorena Meléndez-Illanes, Cristina González-Díaz, Carlos Álvarez-Dardet

**Affiliations:** 1 Department of Nutrition and Dietetics, Faculty of Pharmacy, University of Concepción, Concepción, Chile; 2 Communication, Food and Consumption Research Group (FOODCO), Alicante, Spain; 3 Department of Communication and Social Psychology, University of Alicante, Alicante, Spain; 4 Faculty of Health Sciences, University of Alicante, Alicante, Spain; 5 Public Health Research Group, University of Alicante, Alicante, Spain; 6 Biomedical Research Center in Epidemiology and Public Health Network (CIBERESP), University of Alicante, Alicante, Spain

**Keywords:** Social networks, Advertisements, Advertising, Marketing, Foods, Food advertising

## Abstract

**Background:**

This work is aimed to describe the recent scientific literature developed in the food/beverage sector and social networks aimed at children and adolescents, as well as current regulations.

**Methods:**

A rapid review of the studies on advertising and social media aimed at children, published from 2010 tp 2020 is carried out, following the established inclusion criteria. In addition, the regulations in the countries in which the studies were carried out were also reviewed.

**Results:**

Of the 573 articles, 7 met these criteria. The great attraction of unhealthy foods for children is observed, who remembered and recognized a greater number of unhealthy food brands and, by extension, the ability to influence of communication in social networks. Each country has its own self-regulation, two (Ireland and USA) have legislation on children’s food advertising, and Australia has legislation that applies only to open television. However, none of the analyzed countries have specific regulations on food, children and media advertising.

**Conclusions:**

Given the fact that there is evidence about the great attraction that social networks suppose for the child, that they are a stimulus for the consumption of food and that many of these foods are harmful to their health; we should work in two directions: 1) Promote public policies based on promoting healthy habits among minors; and 2) Monitor and implement regulations in commercial communication social media.

## Introduction

The marketing of foods high in saturated fats, trans fats, free sugars and/or salt (HFSS) aimed at children is omnipresent and is carried out through multiple channels and in different media [1, 2]. It is evident how almost all marketing promotes unhealthy foods, presenting a distorted image of consumption and normalizing their intake [3–5]. Both official bodies such as the WHO as well as recent academic studies, raise the alarm by consoodering advertising as a potential catalysu of obesity and overweight [1–7].

On the other hand, it should be emphasized that the communicative context has changed. In this way, although television is still predominant on the part of the food companies to address children, the online environment has notably increased its presence in this sector and for this specific population [8, 9]. It is noteworthy how children across Europe use digital media avidly and increasingly [10]. According to the EU Kids report [11], of the 19 participating countries, it is confirmed that in 11 of them, more than 80% of children between the ages of 9 and 16 access the Internet at least once a day using their mobile phones. This situation is transferred to the rest of the continents, as shown by studies carried out by Rummo et al., 2020 [8] for the US context; and Freeman et al., 2014 in the US, Canada and Australia [12]. Following the pathway of traditional media, it can be observed how the unhealthy food is also moving to the digital landscape [10, 13, 14]. Thus, it is precisely the food brands that exert the greatest communicative pressure [15, 16]. Within this new and changing digital environment, the use of social networks by minors is strongly entrenched [17]. In the US, 85% of children use YouTube, 72% Instagram, and 69% Snapchat [18].

Similar data can be found in the Australian context, in which almost half of the children use regularly the social between the ages of 8 and 11use regularly the social network YouTube [19]. In line with these results the last report on the digital habits of children (2020) carried out based on the consumption of applications in three of the main markets.US, United Kingdom and Spain- throughout 2019 and 2020; shows how in 2020 the average time spent on social applications increased by 100% on all platforms, with TikTok, Instagram and Snapchat being the most popular.

From the communicative point of view, the digital context offers novel and persuasive advertising designs that further aggravate the difficulty of detecting and understanding them by children and adolescents [3, 13]. The prevalence of advergames in websites aimed at children has been confirmed [20]. These online games, where the protagonist is the brand make difficult to discern what is advertising and what is game [21]. On the other hand, the negative influence of influencers when recommending unhealthy foods has also been highlighted in studies such as that carried out by [4, 13]. Within the influence marketing, the role of Youtubers and their clear influence on the consumption of food products that are not recommended from the point a health point of view should be noticed [15, 22–25]. In this sense, there are already studies showing how kid influencers (3 to 14 years) on Youtube promote food and/or drinks linked to unhealthy branded items [26]. However, there are also studies that show the opposite: how effective influencers can be on children to encourage healthy eating [27].

One of the most widespread recommendations in the documents prepared by the WHO to try to raise awareness about the need for a healthy diet and decrease the marketing of HFSS products has been the self-regulation as a complement/alternative to regulation [1]. At this point, the scientific community is critical about the practical absence of consensual regulation worldwide in this area, with the option of self-regulation being the predominant route [4, 17]. In the study published by the WHO in 2018, a summary of the main restrictions at European level was shown, related to the marketing of foods in traditional media. In this sense, the United Kingdom in 2007 was the first European country to implement legislation in this area. It was followed by countries such as Ireland, Portugal or Norway. However, in Europe the notion of self-regulation prevails [7, 28].

In many of these countries that have chosen this pathway, they do so under the umbrella of the EU Pledge, a voluntary initiative by the main food/beverage companies, in order to change the way in which these products are advertised to children [7]. Paradoxically, studies show how the self-regulation pathway is ineffective [29]. At this point, there are already experts that alert about the difficulty for the industry to regulate itself, because its essential premise is to create profits [30]. In addition, the studies show the loopholes of the codes in progress [2, 30, 31].

Emphasis has been placed on how the main problem to be solved would be the excessive exposure of children to unhealthy products [30]; since it is curious that unhealthy food is not restricted (despite the recommendations), though other types of products such as alcohol or dietetics are [17]. Furthermore, in the new communicative environment the practical illegality of the new strategies used is evident [15]. In a recent document published by the WHO, the focus has already been placed on monitoring the type of food marketing aimed at children, with special emphasis on digital marketing. Aspects such as restricting the digital communication of harmful products for children and adolescents through the CLICK tool, studying the impact of influencer marketing or how to monitor this type of strategies in the online environment, have been the topics covered in this working document [29]. In this sense, it has been observed that although the WHO prefers to recommend instead of prohibiting, the PAHO (the WHO office in the Americas) directly chooses to prohibit [32]. In this way, there is little unanimity in the policies to be followed between regions in the face of a common problem.

Despite the fact that food advertising aimed at children is increasingly focused on the online environment [13] and its consumption has increased [17], recent studies continue to focus their attention on the television environment [1, 21, 30, 31, 33]. At this point, the critical systematic review stated out by Smith et al. (2019) can be emphasized [34]. This is related to studies carried out until 2018 were focused on marketing techniques used to promote food products aimed at children. Of the 71 studies selected, 38 were focused on television and movies. Regarding the digital panorama, interest in the study of advergames is observed, though only 2 articles focused on the Internet in a generic way were observed. On the other hand, although it be observed that studies on the digital environment are scarce compared to those focused on traditional media such as television, they are increasing. It is also observed that the scientific literature is scarce regarding the study of a channel that is currently on the rise: social networks. At this point, the article on scientific literature that addresses the influence of social networks on food from 2015 to 2020 is highlighted. The authors notice the enormous attraction that this age group feels for unhealthy foods advertised through this channel; as well as that the influencer strategy is the predominant [35].

This knowledge gap, which represents the growing but still scarce literature regarding the food/beverage advertising directed to children through social networks is opposed to the imminent health consequences that exposure to said advertising could be generating, considering Internet use and access to this technology is growing and not sufficiently supervised.

With this starting point, this work is aimed to describe the recent scientific literature developed in the food/beverage sector and social networks aimed at children and adolescents, as well as current regulations. Specifically, it is intended: 1) To determine the studies on social networks and food brands aimed at children; 2) Observe the methodology used, as well as the population under study; 3) Analyze the main results shown by the studies; and 4) Study the current regulations on the field that contextualize the works under study.

The purpose of the current study is to offer a recent and current work on the subject, which helps the scientific community to open new lines of research, as well as to implement further measures that can counteract the potential harmful effects pf food advertising in social networks aimed at children.

## Materials and methods

For this study, a rapid review was carried out to offer agile and updated information about the status of the available literature regarding food/beverage advertising in social networks aimed at children and adolescents. The rapid review is based on a simplified approach which is aimed to synthesize evidence in a timely, dynamic and up-to-date manner. Following [36]: “A rapid review a system of knowledge synthesis that accelerates the process of conducting a traditional systematic review by simplifying or ignoring specific methods to produce evidence for stakeholders in an efficient manner in the use of resources”. Although some authors have argued that there is no established method for its attainment, there are several common approaches that speak of its methodological rigor for the purpose at hand. These include requests for timely evidence for decision making, and even to address urgent and emerging health issues that are considered to be considered of high priority [37, 38].

### Search profile

The databases consulted were Web of Science, Pubmed and Scopus. The search was carried out by the first author during September and Octuber, 2020.

The search equation was: “social networks” AND “advertisements” OR “advertising” OR “marketing” AND “foods” OR “food preferences” AND “food advertising” (MeSH Terms).

### Inclusion and exclusion criteria

The corresponding studies carried out in humans were selected, in the children and adolescent age groups, published in English and Spanish, in which some component of food and beverage marketing through social networks was evaluated. The period was limited to the last 10 years, from October 2010 to October 2020, This, considering both birth and evolution of social networks over time [39].

All initially preselected documents were evaluated by the lead author of this article. Disagreements on whether to include some of the studies were resolved by reaching a consensus between three authors.

Studies that could not meet the aforementioned criteria and that corresponded to studies that evaluated the marketing influence through other methods were excluded. Likewise, studies that could not evaluate the influence of marketing on children and adolescents, studies that evaluated the influence of other products, studies that evaluated a brand, studies that evaluated the influence of children´s characters and others (carried out on animals, studies that evaluate other effects, etc.) were also excluded.

### Analysis of the scientific literature and current regulations

From the studies finally selected for the rapid review, the authors LM and CG collected information on the following variables: authors, name of the journal, year of publication, sample size, design, country of origin of the study, outcomes, main conclusions and future line of research. Together with the analysis of scientific literature, the authors LM, CG and CA performed an analysis focused on two parameters: 1) To observe if the analyses are referred to the existing regulations in the field (either in the theoretical framework or applied, observing its compliance; or 2) analyze the existing regulations (both legislations as self-regulation) taking into account the geographical context of the selected studies.

The analysis included a review of websites of agencies and regulatory entities of the countries of origin of the publications found to inquire about the regulations and/or self-regulation of each country. Considering each country, the following information was collected according to the following variables: 1) if there is specific legislation/self-regulation about advertising in social networks. Once the existence of legislation/self-regulation was detected, an analysis of the regulations was carried out, as the case may be, taking into account the following items: 2) denomination; 3) age range covered; 4) media/formats served; 5) types of foods you consider; 6) how it is controlled; and 7) other aspects of interest to be considered.

## Results

**Fig. 1:**
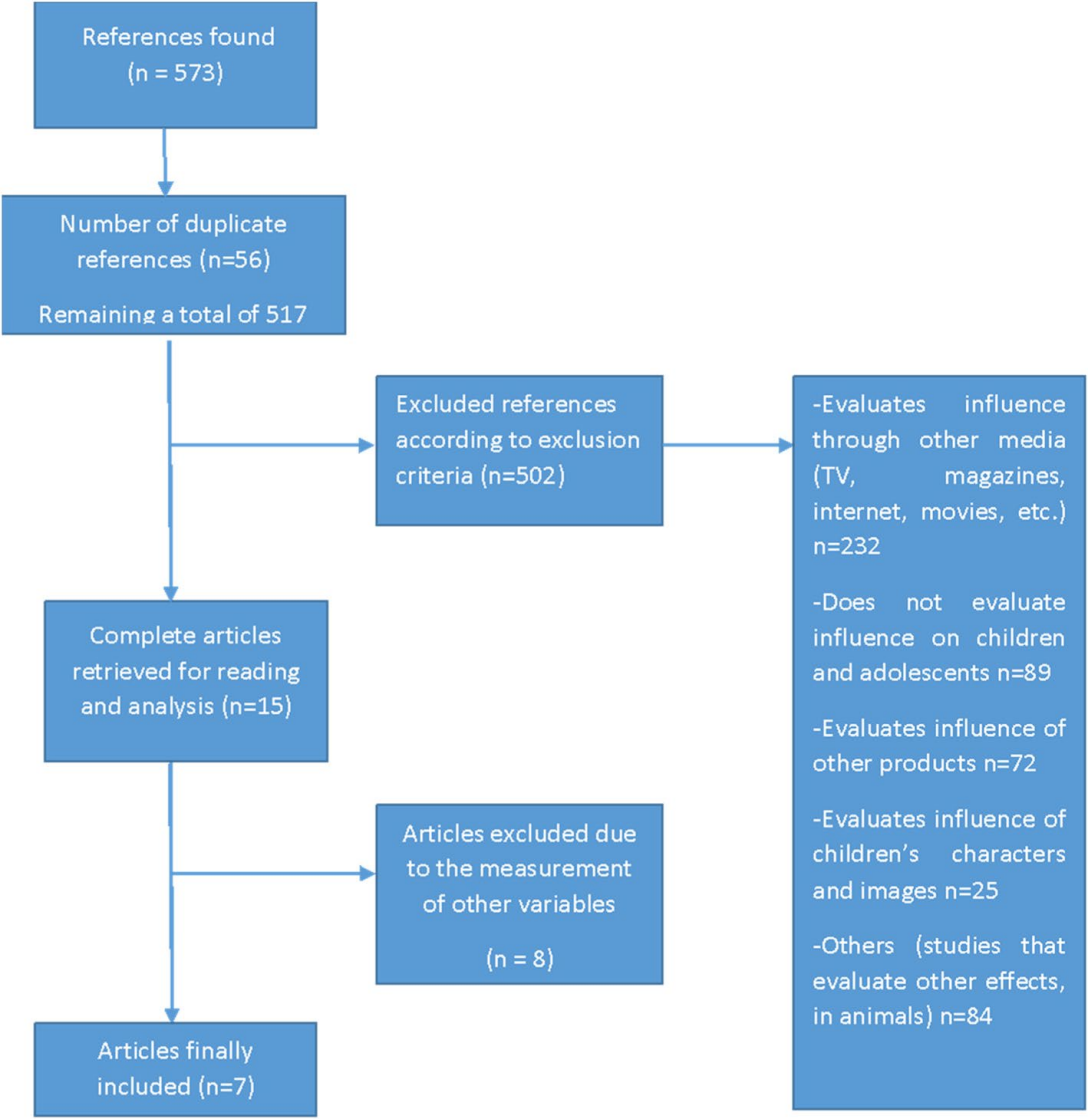
Diagram of the systematic review process

In the initial search, 573 articles were found, of which 66 were duplicates, after their review, 502 articles were excluded, which corresponded to 232 articles that evaluated influence through other media (TV, magazines, internet, movies, etc.). Other 69 studies that evaluated influence in other age groups; 72 studies that evaluated influence in other products; 25 studies that evaluated influence of children’s characters and images; and 84 studies that evaluated other effects that evaluated other effects in animals. Etc. (Fig. 1).

A total of 15 studies were selected for a complete review. Of them, 8 corresponded to studies that measured other variables, leaving a total of 7 studies that met the inclusion criteria. The oldest study was published in 2014 [12] and the most recent 2020 [6, 8, 40]. Three of the articles were carried out in Australia [12, 41, 42], two in the US [6, 8], one in New Zealand [43] and one in Ireland [40].

Regarding the years of publication, three articles were published in 2020, two in 2016, one in 2017 and one in 2014.

Regarding the ages at which the studies were carried out, in those that included participants corresponded mainly adolescents, two were carried out in children between 13 and 17 years old (29%), one of 6 to 17 years old (14%), and one from 11 to 17 years old (14%).

When analyzing the results of the search, it is observed that three (43%) of the studies use surveys conducted on adolescents regarding advertising on social networks [6, 40, 42], two (29%) correspond to the analysis of internet traffic in a given period (years 2014 and 2019) [8, 43], one (14% corresponds to an audit of media for three food and beverage brands (McDonald’s, Coca Cola and Cadbury Dairy Milk) in Facebook, websites and mobile phone apps) [41], and one (14%) to a content analysis of the marketing techniques used through Facebook [12] (Table 1).

**Table 1:** Summary of the studies included in the systematic review, characteristics, main findings and conclusions

**Number of articles**	**Authors/** **year**	**Name of study**	**Journal and citation**	**Design**	**Nationality**	**Number of participants**	**Age**	**Outcomes**	**Results**	**Future line of research**
**1**	Fleming-Milici & Harris/2020 Adolescents’ engagement with unhealthy food and beverage brands on social media [6]	Adolescents’ engagement with unhealthy food and beverage brands on social media	Appetite	Cross-sectional online survey of	USA	1564 adolescents	13–17	Teen engagement on social media with food/beverage brands, sociodemographic differences in the level of engagement, and relationships between engagement and screen time	70% of teens reported engaging with any food/beverage brand on social media (between 1 and 48) and 35% engaged with more than 5 brands. About half reported interacting with fast food brands (54% of participants), sugary drinks (50%), sweets (46%), and snacks (45%), while only 7% reported interacting with all other food categories. / Beverage brands	Future research should assess the activities that adolescents engage in when they spend time on other screens. The results of this study also suggest that time spent on TV viewing and other types of screens should not be combined into a single media use variable Research is also needed to understand the underlying reasons for high levels of social media brand engagement by some groups, including Black, less-acculturated Hispanic, and younger adolescents
**2**	Murphy et al./2020 [40]	See, Like, Share, Remember: Adolescents' Responses to Unhealthy-, Healthy- and Non-Food Advertising in social media	Int J Environ Res Public Health	Questions answered based on the free recall of the brand and recognition	Ireland	151 adolescents	13–17	Teen Responses to Unhealthy, Healthy, and Non-Food Advertising on social media	Compared to unhealthy foods, 5 out of 6 measures: teens were more likely to want to "share" unhealthy posts; rated peers more positively when they had unhealthy posts in their feeds; recalled and recognized a greater number of unhealthy food brands; and seen unhealthy advertising posts for longer	Future areas of exploration are links between social responses to food marketing (sharing and peer assessment) and consumption patterns
**3**	Rummo et al./2020 [8]	Examining the Relationship between Youth-Targeted Food Marketing Expenditures and the Demographics of Social Media Followers	Int J Environ Res Public Health	Purchase of Demographics Pro data to characterize the demographics of Twitter and Instagram users who followed 27 of the most hyped fast food, snack and beverage brands in 2019	USA	-	-	Determine how many teens follow food/beverage brands on Instagram and Twitter and examine associations between the brands' youth-targeted marketing practices and teen follow-up percentages	An estimated 6.2 million teens followed the selected brands. A higher% of teens followed the accounts of the selected brands (9.2%) compared to any Twitter account (1.2%) (p < 0.001), but not Instagram. A higher% of teens followed sugary beverage brands (7.9%) versus low-calorie beverage brands (4.3%) on Instagram (p = 0.02), but we observed the opposite pattern for adults on Twitter and Instagram	Future studies should explore similar research questions using YouTube, which has a higher percentage of adolescent users relative to Instagram and Twitter
4	Vandevijvere et al./2017 [43]	Unhealthy food marketing to New Zealand children and adolescents through the internet	New Zealand medical association	Analysis of internet traffic data from January 2014 was acquired from AC Nielsen to identify the largest amount of Popular websites (n = 110) among children and adolescents	New Zealand	70 food brands	6 a 17	Measure the impact of the type of publication (advertising, fan, events, information and promotion) on two interaction metrics: likes and comments	Compared to traditional media, the Internet allows food vendors to use techniques to interact directly with children. While the range of marketing techniques and features identified on food brand websites was extensive, the most popular websites among children and teens were non-food-related, and the scope of food marketing on those websites was low. In addition, it is recommended to evaluate the marketing of food to children through social media and other digital media	Additional assessment of food marketing to children and adolescents through social and other digital media is crucial, since companies may have shifted their marketing efforts to those new media
5	Thaichon & Quach/2016 [42]	Online marketing communications and childhood’s intention to consume unhealthy food	Australasian Marketing Journal	Qualitative, inductive approach to data collection using a sample of Australian children who use social network sites and their parents	Australia	30 adolescents	11–16	Impact of online advertising on social networking sites with the intention of children to consume fast food in Australia	It was found that fast food ads on social media sites could manipulate the young audience in terms of likelihood of purchase, opinions about fast food, and eating habits The results of the interviews indicated that peer pressure is an important element of online communications on social media. By showing their ads to a group of young consumers, companies can create a sense of socialization and associate their product with a community. This study is one of the first to consider general aspects of children's perception of marketing communications on social media sites in the context of the fast-food industry	A possible opportunity for future research is to investigate whether online communications on social networking sites can alter children’s actual behavior. In addition, it would be interesting to examine the changing patterns of children’s attitude and behavior toward fast food through longitudinal loyalty
6	Boelsen-Robinson et al./2016 [41]	Digital marketing of unhealthy foods to Australian children and adolescents	Health Promotion International	Media audit for three food and beverage brands / Facebook, websites and mobile phone apps from three brands	Australia	(McDonald’s, Coca-Cola, and Cadbury Dairy Milk)	–	New Media Audit for Three Food and Beverage Brands in Australia	it was found that although all promotional activities technically complied with self-regulatory codes (usually due to media-specific age restrictions) it appeared to employ unhealthy food or beverage marketing aimed at children. Brands are using engaging content through new media targeting children and teens to promote unhealthy foods and beverages	Future studies should examine the impact of these media on purchasing, consumption and preferences of youth. Furthermore, the unique aspects of marketing via new media, such as viral marketing, should be examined to determine their effect on behaviour
7	Freeman et al./2014 [12]	Digital Junk: Food and Beverage Marketing on Facebook Becky	American Journal of Public Health	Content analysis of the marketing techniques used / Facebook	Australia	27 Facebook pages	–	the quantity, scope and nature of energy-dense and nutrient-poor food marketing on Facebook was assessed	By using the interactive and social aspects of Facebook to market products, energy-dense, nutrient-poor food brands leverage users' social media and expand the reach and personal relevance of their marketing messages	Future studies should look at young adults. this group appears to be a highly desirable target population for food marketing, and limited research, resources, and policy action have been directed in this age group. Emerging adulthood (ages 18 to 25) is largely overlooked in establishing long-term health behavior patterns

Among the results found in the articles, the study carried out in the US stands out [6] where 1,564 adolescents were surveyed. Of them, 70% responded that they participated with at least one food or beverage brand through the interaction on social networks. A third of the sample (35%) responded that interacted with 5 or more brands, and 50& responded that interacted with social networks of sugary brands, beverage brands, candies and snacks (with an average of approximately 2 brands per category).

In another of the selected studies, carried out in Ireland [40], which examined adolescents' attention, memory, and social responses to advertising posts, including interactions between product types and source of the publications, including interactions between types of products and source of the publications, it was observed that the adolescents were found to be more likely to want to “share” unhealthy publications (in 5 of 6 measures). In addition, they rated their peers more positively when they had unhealthy posts in their feeds; recalled and recognized a greater number of unhealthy food brands.

Another aspect analyzed was future lines of research declared by the authors in each article (Table 1). It can be seen that all the studies mention the need for future research in aspects such as evaluating the activities carried out by adolescents when they spend time on other screens [31], understand the underlying reasons for high levels of brand engagement on social media [8, 40], impact of these media on youth purchases, consumption, and preferences of young people [41].

Another aspect analyzed corresponded to the regulatory agencies and entities of the countries assigned to the selected studies. The following websites were reviewed: Food Standards in Australia and New Zealand [44], Department of Agriculture, Food and the Marine in Ireland and in the FDA [45], and the Department of Agriculture and Federal Trade Commission [46]. It is observed that of the four countries to which the selected articles are geographically ascribed, all of them have specific self-regulation about food advertising aimed at children, though only two (USA and Ireland) complete it with legislation in this regard. On the other hand, Australia has legislation but only applied to open television (Table 2). It can be observed that none of the agencies under study has specific food advertising through social networks aimed at children and adolescents. The concept of digital media includes platforms that have child-directed content such as YouTube (USA) virtual, interactive and Split-screen advertising (Ireland).

**Table 2:** Analysis of legislation/self-regulation

	**USA**	**Ireland**	**New Zeland**	**Australia**
**Legislation**	In 2009, bipartisan legislation directed the creation of the Interagency Working Group (IWG) on Food Marketed to Children to curb the marketing of unhealthy foods for children aged 2–17 years old	S.I. No. 331/1991—Health (Foods For Particular Nutritional Uses) Regulations, 1991 No mention of legislation on commercial communication of food aimed at children	No specific legislation is observed	Children’s Television Standards The Children’s Television Standards 2009 (CTS), applying to free-to-air television, is the only government regulation dealing specifically with advertising to children. The Australian Communications and Media Authority (ACMA) developed the CTS under the Broadcasting Services Act 1992 (Cth). Compliance with the CTS is a licence condition for all free-to-air television broadcasters and ACMA is responsible for its administration and enforcement The CTS only applies to advertising on free-to-air television and not to other types of media
**Self-regulation** -Denomination (year)	**- Children Advertising Review Unit (CARU) (1974)** The nation’s first Safe Harbor Program under the Children’s Online Privacy Protection Act (COPPA) helps companies comply with laws and guidelines that protect children from deceptive or inappropriate advertising and ensure that, in an online environment, children's data is collected and handled responsibly **- Children’s Food and Beverage Advertising Initiative in 2006 (CFBAI)** Was created to improve the landscape of food advertising to children under age 12. Under CFBAI, participants voluntarily commit that, in advertising primarily directed to children, they will either not advertise foods or beverages to children at all or advertise only products that meet CFBAI’s strict Uniform Nutrition Criteria	30th June 2021: **The Advertising Standards Authority for Ireland (ASAI)**, the independent self-regulatory body committed to promoting the highest standards of marketing communications in Ireland, has unveiled new rules relating to the advertising of High Fat, Salt and Sugar (HFSS) products which will be incorporated into the Food and Non-Alcoholic Beverages Section of the **ASAI Code** No medium can be used to advertise HFSS products if more than 50% of its audience is under 15 years of age and restrictions apply to the percentage of HFSS marketing communications that can be carried by each media format	The advertising industry is self-regulated through the **Advertising Standards Authority (ASA)**, with junk food marketing towards children falling under the agency’s voluntary **Children and Young People’s Advertising Code**, which is due for its five-yearly review	Food advertising to children (through television and other media) is self-regulated to a limited extent by the Australian Association of National Advertisers (AANA) under its codes of practice, **the Code for** **Advertising & Marketing Communications to Children (Children’s Code)** and **the Food & Beverages Advertising & Marketing Communications Code (Food & Beverages Code)** The Australian Food and Grocery Council (AFGC) introduced two voluntary codes to restrict food advertising to children. **The Responsible Children’s Marketing Initiative (RCMI)** applies to food and beverage advertising (not including fast food advertising) ‘directed primarily to’ children under 12 by food companies that are signatories. **The Quick Service Restaurant Industry Initiative for Responsible Advertising and Marketing to Children (QSRI)** applies to fast food advertising ‘directed primarily to’ children under 14 by fast food companies that are signatories These voluntary codes purport to ensure that food advertising ‘directed primarily to children’ represents healthier choices but the weak provisions provide very little protection The codes are administered by the ASB
-Age scope covered	Children under age 12	children under the age of 15	Definitions for the Purposes of this Code “Children” means all persons below the age of 14 years “Young People” means all persons who are at least 14 years but under 18 years	The QSRI and RCMI only restrict unhealthy food advertising content ‘directed primarily to children’ (Under 14 for the QSRI and under 12 for the RCMI)
-Regulated media/formats	The Core Principles cover child-directed advertising on TV, digital and mobile media (websites, video and computer games, apps, YouTube, product placements and integrations, and influencers), radio, print, and word of mouth	The Code covers commercial marketing communications and sales promotions in all media in Ireland including digital web, social, mobile, in-game ads, influencer marketing (user-generated commercial content), print, outdoor, radio, TV, leaflets/brochures, SMS/MMS, cinema, and direct marketing	This Code applies to all advertisements that target children or young people, whether contained in children’s or young people’s media or otherwise. In determining whether this Code is applicable, the Complaints Board will make an evaluation based on context, medium, audience and product or service This Code does not apply to product packaging, bona fide news, reviews, editorial and broadcast programmes	TV programs or digital media for which children constitute more than 35% of the audience
-Types of foods that are prohibited	Foods advertised to children by CFBAI participants must meet CFBAI’s Uniform Nutrition Criteria, which set limits on calories, saturated fat, sodium, and added sugars, and minimum requirements for important food groups and key nutrients	Food & Non-Alcoholic Beverages	Occasional food or beverage products	Food & Non-Alcoholic Beverages
-How it is controlled	CFBAI monitors and evaluates the participants’ compliance with their pledge commitments, and companies also submit annual self-assessments. CFBAI publishes an annual report on compliance and progress	The new restrictions will come into effect on 1st December 2021 and ASAI will be working with media and advertisers to ensure the successful rollout of the rules. To facilitate this, ASAI will be taking a very practical and staged approach to their implementation They will be proactively monitoring this area and, for the first six months after the effective date, will add any complaints that may be received into their monitoring structure, using them as a form of intelligence gathering	The Advertising Standards Complaints Board and the Advertising Standards Complaints Appeal Board are the final judges of the interpretation of the Codes	Compliance with the voluntary codes is not monitored; the system relies entirely on complaints from the public to identify breaches

In a generic way, they are referred to it countries such as Australia, where restrictions on marketing mainly aimed at children are discussed without specifying whether it is in the online or offline context. However, it is specifically mentioned that other forms of communication are not strictly aimed at children, as for example, the information present in the On-Pack Nutrition Labelling. New Zealand applies its code to all advertising aimed at children, without specifying a particular or concrete channel. On the other hand, there is a lack of consensus on what age the codes are assigned to, and by extension, what is meant by a minor. Although in the US speak of children under 12 years of age, establishing reinforced age bands for those under 6, 13 and 15 years of age; New Zealand defines children as those under 14 and “Young People” as those under 18. It is worth noting the case of Australia, county where a different age range is established depending on the document. Thus, according to the Responsible Children´s Marketing Initiative (RCMI), which is applied to food and beverage manufacturers, children are defined as under 12 years of age. However, according to the Quick Service Restaurant Initiative for Responsible Advertising and Marketing to Children (QSRI), which is applied to fast food chains, minors are those under 14 years of age. Regarding the types of foods and/or beverages covered by the regulations, a common link is observed: to regulate commercial communication with respect to those foods not recommended in the diet of children due to their high content of sugars, fats and / or salt (HFSS).

Finally, if the measures to control compliance with current regulations and, by extension, the corresponding penalties are considered, a series of degrees can be established. On the one hand, there would be countries like the US, which issues public reports, and which can notify the regulatory agencies to proceed with a sanction in the event of non-compliance. On the other hand, New Zealand, through the body called *The Complaints Board* the cases are evaluated, and an opinion is established. Together with these postulates, in the analyzed texts, citizen help is insisted on to formulate complaints (Ireland and Australia).

## Discussion

Although the studies found are scarce and the designs are diverse, significant outcomes can be found, such as the study carried out by Rummo et al. [8], which determined how many adolescents follow food/beverage brands on Instagram and Twitter. Additionally, the associations between marketing practices aimed to adolescents were examined. Differences were observed in the percentages of adolescents who followed brands of sugary drinks compared to brands of low-calorie drinks. The result was a higher percentage of teenagers following sugary drink brands versus low-calorie drink brands on Instagram. Authors such as Jiménez-Marín et al. (2020); Tatlow-Golden & Garde (2020) and Gascoyne et al. (2021) pointed out how the companies spend more in promoting sugary beverages instead of low-calorie ones [3, 4]. Hence, inevitably, the probability that children are exposed to this type of food is greater. For this reason, the high number of children that follow HFSS product brands should not be underestimated, since it corresponds to an important public health problem. There is a correlation between the consumption of this type of beverages with the onset of Type 2 diabetes mellitus and weight gain [1, 7, 8, 31].

Another study included in the review is that carried out by Freeman et al. (2014) analyzes 27 food and beverage Facebook pages (sugary beverages, ice cream, chocolate and fast food) most popular in Australia [12]. This study identified generalized marketing techniques, often unique to social networks that could increase consumer interaction and engagement, and even facilitate the direct purchase of the product.. The study concludes that the use of these interactive and social aspects of Facebook to market these products is common, food brands capitalize on the social networks of the different users and expand the reach and personal relevance of their marketing messages. These results are complemented by Boelsen-Robinson et al. (2016), who performed a new media audit of for three food and beverage brands marketed in Australia and well known worldwide (McDonald’s, Coca-Cola and Cadbury Dairy Milk) [41]. Here, promotional activities were found, which seemed to use a series of marketing strategies with the frequent use of an indirect association of products, participation techniques and branding. From these results, it is inferred that brands are using engaging content through new media aimed at children and adolescents to promote HFSS products. On the other hand, it can be observed that the figure of the influencer is becoming a strategy used by food brands to promote their products [4, 13]. However, the foods promoted by influencers are far from those that promote healthy eating. In this sense, given the limitations of self-regulatory codes in the context of digital media, all strategies should be focused on reducing the exposure of children and adolescents to the marketing of HFSS products through these dissemination channels. The conclusions of these works are in line with what has been stated by Tatlow-Golden & Garde (2020), who raised the need to comprehensively address the protection of the rights of all children against harmful marketing [4].

Regarding the response of adolescents, one of the studies compiled by Murphy et al. (2020) [40] proved the interactions in Facebook. Results showed that unhealthy foods ads evoked significantly more positive responses compared to healthy foods, in 5 of 6 measures. Adolescents were more likely to want to share unhealthy publications and rated their peers more positively when they had unhealthy publications in their networks. In addition, they remembered and recognized a greater number of unhealthy food brands and the interactions with peers, celebrities and companies were greater with unhealthy food advertising. The fact that adolescents are more likely to remember unhealthy food is a finding also described in a recent review published by Kucharczuk et al. (2022) [30]. These results are also related to those found by Thaichon & Quach (2016) [42], who performed an interview with quantitative approach to 30 Australian children who used social networks and their parents. Among the results found, it is highlighted that fast food ads on social networking sites could manipulate the young audience in terms of purchase probability, opinions on fast food and feeding habits. A worrying element was also evidenced: group pressure as an important element of online communications through social networks. Thus, by having ads that create interaction with a group of young consumers, companies can create a sense of socialization and associate their product with a community.

In the document *Tackling food marketing to children in a digital world: trans-disciplinary perspectives* published by the HO, there is a reference to the importance of reducing the exposure of children and adolescents to all forms of HFSS food marketing, focusing on digital media, mentioning that the advertising in these media is increasing. Furthermore, the report points out that brands and marketers are not only remarkably successful, but also amplify the effects of marketing HFSS food [10]. This is consistent with what was posted on the Facebook page stating that social media marketing amplifies the effects of broadcast marketing, increasing target audience reach, ad memorability, brand bonding, and likability to a greater extent. than television alone. [47]

On the other hand, it should not be forgotten that, compared to conventional advertising, commercial communication in the digital context is more subtle and creative, which increases the capacity for persuasion and the difficulty of detecting its commercial nature, especially for adolescents [3, 13]. In addition, they are being offered attractive “information” about an aspect that directly concerns their health: food. In this sense, the responsibility of the parents should also be appealed to. It is necessary that they know the digital environment that surrounds their children; since most of them are not aware of the effects of advertising in a generic way, and of food, specifically, on social networks. This becomes relevant since accompanying and guiding children in the formation of healthy eating habits is not only a search for a solution to malnutrition due to excess, but also a method to achieve a healthier society.

Regarding the regulation, of the four countries analyzed all have self-regulation, though only the US and Ireland have legislation about on food communication aimed at children. On the other hand, Australia only has legislation related to open television. Thus, following Tatlow-Golden & Garde, 2020 [4] y Sacks & Looi, 2020 [17] it can be observed how the predominant path is that of the self-regulation. However, the analysis indicates how, with the exception of the US and Ireland, no express mention is made on platforms such as social networks in which food brands exert increasing pressure [15, 48]. Therefore, they are firmly entrenched among the media diet of children and adolescents [17]. It is observed how the regulation is focused on conventional media or in the advertising communication without specifying the channels and formats of the digital context. Furthermore, there is a cleat lack of consensus about should be protected when referring to minors, because depending on the country, there are variations in the age ranges.

There are already authors who highlight the inefficacy of the self-regulation [49], because the regulation is rather limited to television advertising and the marketers shift their investments to other platforms. Consequently, HFSS food marketing restrictions therefore need to implement policies to protect online advertising need to be implemented [50].

This focus on conventional media is also evident in the academic studies reviewed on the binomial food advertising aimed at children and minors. The research is focused on television versus the Internet, with no studies specifically focused on social networks having been detected [34].

The inappropriate use of the “marketing” concept has also been accepted, since the studies are referred to it to mention advertising. However, marketing assits to much more: promotion, product, distribution and price. Without going any further, when making the search equations, although our focus was advertising, we had to add the marketing item when we detected that many of the studies of interest use both terms interchangeably. However, this terminological problem in the mention used in the normative texts analyzed, in which there is a consensus on the term referred on the regulations: advertising or commercial communication. It is important to notice that marketing and advertising are two different terms, with advertising being a part of the former. Failure to establish this clarity can cause difficulties in determining both the object of study and the corresponding regulations.

### Policy implications

The present review presents a distinct paucity of studies on the subject. Among the most relevant results, the potential influence of social networks on the consumption patterns of children stands out, and the predominance of undesirable food products in their diet.

On the other hand, it can be observed that regulations which standardize the advertising of food/beverage aimed at children through social networks is practically inexistent. To this fact, the aggravating factor that various authors point out must be added: the current self-regulatory measures are not sufficient.

These results should encourage the authorities to promote food policies aimed at caring for the health of minors, who are currently deliberately exposed to advertising unhealthy or rather harmful food and beverages that only contribute to worsening their feeding habits. Actions based on promoting healthy habits among children and adolescents should also be implemented. In addition, efforts must be redoubled both in the proliferation of more rigid regulatory measures regarding the dissemination of food advertising, especially less healthy ones; as well as seeking more decisive sanctions that make brands understand their responsibility with respect to the media and food diet of a vulnerable public.

### Proposal for a research agenda

This work highlights the urgent need to stimulate research in this area of knowledge, given the limited of studies. Based on the results obtained, the research agenda should be directed towards the following lines of research:

- Applied works in which the potential influence between online media, specially social networks, and the consumption/demand of food products by children is observed. Studies have shown how brands promote HFSS products, and it is now time to investigate the potential for influence. Furthermore, this influence capacity should be determined by online media: does the advergaming strategy have the same influence as the use of a social network such as Youtube or Instagram? Within social networks, what influence capacity does the use of influencers have? etc.
- Following on from the previous line, it is important to determine that, once the potential influence of online media on the consumption of foods that are not recommended in children's diets has been measured, this knowledge can be used in a positive sense: using these same communication strategies to promote healthy lifestyles. In this way, exploratory studies along these lines should also be investigated following the parameters of the work of De Jans, S et al. (2021) [27].
- This work shows that there is no consensus in determining the age range for defining the child/children concept. The studies analysed address the 6 to 17 age range. However, despite the fact that we are dealing with a credulous and vulnerable public, most of the empirical literature recognises the vulnerability of the very young (0 to 6 years, 6–8 years) and that the influence of advertising to this group will have a differential impact on older age groups. An empirical study focusing on the impact of advertising on the young people needs to recognize these differential effects and provide evidence along these lines rather than assume that all impacts are equally detrimental.
- The research shows that the existing normative is limited and very generic. There are also initiatives already underway that are on the way to prohibition. As a complementary measure to these legislative policies, work should be encouraged to measure the degree of effectiveness of the restrictions implemented.
- The concept of “children advertising literacy” must prevail above all else. Studies should be carried out on public policies that highlight the actions taken by administrations to promote children's media literacy in relation to these not-so-new media and the advertising that accompanies them.

## Conclusions

The present work highlights the worrying low number of studies that, specifically, address advertising of food and beverages through social networks aimed at children and adolescents. In addition, the specific regulation on this topic is practically anecdotal. Inquiring into this fact, the WHO, already in 2016, expressed its concern about the small number of existing studies that revealed sufficient academic evidence between exposure or impact and effects on the digital marketing of HFSS foods in minors; and from them, base new regulations [10].

With this starting point there is an urgent need for the proliferation of works related to the advertising communication of food and beverages aimed at minors in the digital context, in a generic way and in the landscape of social networks. It is also interesting to study, on the one hand, if the existing regulations are complied with; as well as stimulating improvement proposals adapted to the communicative particularities of these not so new online channels.

Finally, it can be observed that there are already governments such as the British one, which advocates the route of banning HFSS food advertising aimed at children in all media. Other, such as the Spanish government although it have recently been in favor of prohibition in the legal field, already have initiatives such as the “Defend me” campaign, focused on the prohibition of the advertising of unhealthy foods aimed at the child population. Along with these measures, of which it is necessary to observe their degree of effectiveness and scope, it is considered that public policies that promote healthy diets among children and adolescents should be taken. On the one hand, because a healthy eating is considered a human right [15]. On the other hand, from the scientific community it is observed that the intake of HFSS food can be a potential catalyst of obesity and it must be taken into account that this type of non-communicable diseases imply a medical cost.

## Data Availability

All data generated or analysed during this study are included in this published article.

## References

[1] Landwehr SC, Hartmann M (2020). Industry self-regulation of food advertisement to children: compliance versus effectiveness of the EU Pledge. Food Policy.

[2] Royo-Bordonada MÁ, Rodríguez-Artalejo F, Bes-Rastrollo M (2019). Food policies to prevent obesity and the main non-transmissible diseases in Spain: where there’s a will there’s a way. Gac Sanit.

[3] Jiménez-Marín G, Zambrano RE, Galiano-Coronil A (2020). Food and beverage advertising aimed at Spanish children issued through mobile devices: a study from a social marketing and happiness management perspective. Int J Environ Res Public Health.

[4] Tatlow-Golden M, Garde A (2020). Digital food marketing to children: exploitation, surveillance and rights violations. Glob Food Sec.

[5] Gascoyne C, Scully M, Wakefield M (2021). Food and drink marketing on social media and dietary intake in Australian adolescents: findings from a cross-sectional survey. Appetite.

[6] Fleming-Milici F, Harris JL (2020). Adolescents’ engagement with unhealthy food and beverage brands on social media. Appetite.

[7] WHO. Evaluating implementation of the who set of recommendations on the marketing of foods and non-alcoholic beverages to children. Progress, challenges and guidance for next steps in the WHO European Region. Copenhagen, Denmark; 2018. Available from: http://www.euro.who.int/pubrequest

[8] Rummo PE, Cassidy O, Wells I (2020). Examining the relationship between youth-targeted food marketing expenditures and the demographics of social media followers. Int J Environ Res Public Health.

[9] Bragg MA, Eby M, Arshonsky J (2017). Comparison of online marketing techniques on food and beverage companies’ websites in six countries. Global Health.

[10] WHO. Tackling food marketing to children in a digital world: trans-disciplinary perspectives Children’s rights, evidence of impact, methodological challenges, regulatory options and policy implications for the WHO European Region; 2016. Available from: http://www.euro.who.int/pubrequest

[11] Smahel D, Machackova H, Mascheroni G, et al. EU Kids Online 2020. Available from: www.eukidsonline.net

[12] Freeman B, Kelly B, Baur L (2014). Digital junk: food and beverage marketing on facebook. Am J Public Health.

[13] Radesky J, Chassiakos YR, Ameenuddin N (2020). Digital advertising to children. Pediatrics.

[14] Castelló-Martínez A, Tur-Viñes V (2021). A high-risk combination: obesity, food brands, minors and challenges on YouTube. Gac Sanit.

[15] Tur-Viñes V, Castelló-Martínez A (2021). Food brands, youtube and children: media practices in the context of the paos self-regulation code. Commun Soc.

[16] IAB.Spain. Estudio anual de redes sociales 2021.

[17] Sacks G, Looi ESY (2020). The advertising policies of major social media platforms overlook the imperative to restrict the exposure of children and adolescents to the promotion of unhealthy foods and beverages. Int J Environ Res Public Health.

[18] Anderson, M, Jiang J. Research Associate Aaron Smith, Associate Director Tom Caiazza, Communications Manager 202.419.4372; 2018. https://www.pewresearch.org.

[19] Australian Communications and Media Authority (2013). Like, post, share: Young Australians´experience of social media.

[20] Hurwitz LB, Montague H, Wartella E (2017). Food marketing to children online: a content analysis of food company websites. Health Commun.

[21] Folkvord F, van ‘t Riet J. The persuasive effect of advergames promoting unhealthy foods among children: a meta-analysis. Vol. 129, Appetite. Academic Press; 2018. p. 245–51. 10.1016/j.appet.2018.07.020 30031786

[22] Tan L, Ng SH, Omar A, Karupaiah T (2018). What’s on YouTube? A case study on food and beverage advertising in videos targeted at children on social media. Childhood Obes.

[23] Baldwin HJ, Freeman B, Kelly B (2018). Like and share: associations between social media engagement and dietary choices in children. Public Health Nutr.

[24] Coates AE, Hardman CA, Halford JCG (2020). “It’s just addictive people that make addictive videos”: children’s understanding of and attitudes towards influencer marketing of food and beverages by YouTube video bloggers. Int J Environ Res Public Health.

[25] Coates AE, Hardman CA, Halford JCG (2019). The effect of influencer marketing of food and a “protective” advertising disclosure on children's food intake. Pediatr Obes.

[26] Alruwaily A, Mangold C, Greene T, Arshonsky J, Cassidy O, Pomeranz JL, Bragg M (2020). Child social media influencers and unhealthy food product placement. Pediatrics.

[27] De Jans S, Spielvogel I, Naderer B, Hudders L (2021). Digital food marketing to children: how an influencer's lifestyle can stimulate healthy food choices among children. Appetite.

[28] González DC (2013). Autorregulación en la publicidad de alimentos para niños a través de PAOS: un estudio internacional. Cuadernos.info.

[29] WHO. Regional Office for Europe. 14th Meeting of the WHO European action network on reducing marketing pressure on children, Bern, Switzerland, 8 – 9 May 2019: meeting report. World Health Organization. Regional Office for Europe; 2021. https://apps.who.int/iris/handle/10665/342853.

[30] Montaña M, Jiménez-Morales M, Vàzquez M (2016). Food advertising and prevention of childhood obesity in spain: analysis of the nutritional value of the products and discursive strategies used in the ads most viewed by children from 2016 to 2018. Nutrients.

[31] Fleming-Milici F, Harris JL (2020). Food marketing to children in the United States: can industry voluntarily do the right thing for children’s health?.

[32] Recommendations from a Pan American Health Organization Expert Consultation on the Marketing of Food and Non-Alcoholic Beverages to Children in the Americas. Washington, D.C. 20037, U.S.A.; 2011. https://iris.paho.org/handle/10665.2/3594

[33] Norman J, Kelly B, McMahon AT, Boyland E, Baur LA, Chapman K, King L, Hughes C, Bauman A. Sustained impact of energy-dense TV and online food advertising on children's dietary intake: a within-subject, randomised, crossover, counter-balanced trial. Int J Behav Nutr Phys Act. 2018;15(1):37. 10.1186/s12966-018-0672-6 PMC5897936 29650023

[34] Smith, R., Kelly, B., Yeatman, H., et al. Food marketing influences children’s attitudes, preferences and consumption: a systematic critical review. In Nutrients (Vol. 11, Issue 4). MDPI AG. 2019. 10.3390/nu11040875 10.3390/nu11040875 PMC6520952 31003489

[35] Kucharczuk AJ, Oliver TL, Dowdell EB (2022). Social media’s influence on adolescents′ food choices: a mixed studies systematic literature review.

[36] Hamel C, Michaud A, Thuku M, et al. Defining rapid reviews: a systematic scoping review and thematic analysis of definitions and defining characteristics of rapid reviews. Vol. 129, Journal of Clinical Epidemiology. Elsevier Inc.;2021. p. 74–85. 10.1016/j.jclinepi.2020.09.041 33038541

[37] Garritty C, Gartlehner G, Nussbaumer-Streit B (2021). Cochrane rapid reviews methods group offers evidence-informed guidance to conduct rapid reviews. J Clin Epidemiol.

[38] Tricco AC, Antony J, Zarin W (2015). A scoping review of rapid review methods. BMC Med.

[39] Pew Research Center. (2021, September 1). https://www.pewresearch.org.

[40] Murphy G, Corcoran C, Tatlow-Golden M (2020). See, like, share, remember: adolescents’ responses to unhealthy-, healthy- and non-food advertising in social media. Int J Environ Res Public Health.

[41] Boelsen-Robinson T, Backholer K, Peeters A (2016). Digital marketing of unhealthy foods to Australian children and adolescents. Health Promot Int.

[42] Thaichon P, Quach TN (2016). Online marketing communications and childhood’s intention to consume unhealthy food. Australas Mark J.

[43] Vandevijvere S, Sagar K, Kelly B (2017). Unhealthy food marketing to New Zealand children and adolescents through the internet. NZ Med J.

[44] FSANZ (2021). Foods Standards Australia New Zealand.

[45] Department of Agriculture, F. and the M. gov.ie; 2021. https://www.gov.ie/.

[46] USDA (2021). U.S. Department of Agriculture.

[47] Facebook (2015). Introducing new ways to buy, optimise and measure ads for a mobile world.

[48] Dwivedi YK, Ismagilova E, Hughes DL et al. Setting the future of digital and social media marketing research: perspectives and research propositions. Int J Inform Manag. 2021;59. 10.1016/j.ijinfomgt.2020.102168

[49] Handsley E, Nehmy C, Mehta K (2007). Media, public health and law: a lawyer’s primer on the food advertising debate. Media Arts Law Rev.

[50] Kraak VI, Vandevijvere S, Sacks G (2016). Progrès réalisés pour restreindre la commercialisation d’aliments et de boissons riches en graisses, en sucre ou en sel destinés aux enfants. Bull World Health Organ.

